# Exceptional–point–enhanced phase sensing

**DOI:** 10.1126/sciadv.adl5037

**Published:** 2024-04-05

**Authors:** Wenbo Mao, Zhoutian Fu, Yihang Li, Fu Li, Lan Yang

**Affiliations:** Department of Electrical and Systems Engineering, Washington University, St. Louis, MO 63130, USA.

## Abstract

Optical sensors, crucial in diverse fields like gravitational wave detection, biomedical imaging, and structural health monitoring, rely on optical phase to convey valuable information. Enhancing sensitivity is important for detecting weak signals. Exceptional points (EPs), identified in non-Hermitian systems, offer great potential for advanced sensors, given their marked response to perturbations. However, strict physical requirements for operating a sensor at EPs limit broader applications. Here, we introduce an EP-enhanced sensing platform featuring plug-in external sensors separated from an EP control unit. EPs are achieved without modifying the sensor, solely through control-unit adjustments. This configuration converts and amplifies optical phase changes into quantifiable spectral features. By separating sensing and control functions, we expand the applicability of EP enhancement to various conventional sensors. As a proof-of-concept, we demonstrate a sixfold reduction in the detection limit of fiber-optic strain sensing using this configuration. This work establishes a universal platform for applying EP enhancement to diverse phase-dependent structures, promising ultrahigh-sensitivity sensing across various applications.

## Teaser

A universal platform leveraging exceptional points enhances the sensitivity of ordinary optical sensors.

## INTRODUCTION

Exceptional points (EPs) represent unique spectral singularities of non-Hermitian systems, at which the eigenvalues and their corresponding eigenstates coalesce (*1*–*5*). These degenerate points have been widely observed in diverse physical systems, spanning optical microcavities (*6*–*21*), plasmonic metamaterials (*22*, *23*), photonic crystals (PhCs) (*24*, *25*), acoustics (*26*), electronics (*27*–*32*), elastodynamics (*33*, *34*), and superconducting circuits (*35*). Notably, EPs have unlocked numerous intriguing phenomena in optical systems. For instance, the realization of unidirectional optical metamaterials have been demonstrated through periodic refractive index modulation (*36*, *37*). EP-induced strong chiral behaviors have enabled directional lasing in a whispering-gallery-mode (WGM) microresonator (WGMR) (*19*, *38*). The modification of density of states at EPs has been studied for light sources in PhCs (*39*, *40*) and microresonators (*12*, *41*, *42*). Furthermore, unconventional light control near EPs has been extensively investigated, giving rise to phenomena such as electromagnetically induced transparency (*43*) and coherent perfect absorption (*11*, *17*, *44*). In the past few years, non-Hermitian topological photonics has also attracted considerable attention for introducing more features to optical systems, such as topologically protected light propagation and lasing edge modes with robustness against defects and fabrication imperfections (*45*, *46*).

An appealing feature of EPs is their distinctive sensitivity to perturbations. Generally, in the case of an *N*th-order EP where *N* eigenstates coalesce, the resulting eigenenergy (e.g., resonant frequency for optical microcavities) splitting scales as the *N*th root of the perturbation strength ε (*3*–*5*). In contrast, the splitting is proportional to ε in a system around the conventional degeneracies, known as diabolic points (DPs), at which only the energy levels degenerate while the eigenstates remain orthogonal to each other. The enhancement factor ε^−(*N* − 1)/*N*^, defined as the ratio of the EP-enhanced splitting to the splitting at a corresponding DP, tends to infinity as the perturbation ε → 0. Thus, operating a sensor at EPs can reduce the detection limit for weak signals.

The EP-enhanced optical sensors have been demonstrated for various applications, including nanoparticle detection (*10*, *20*, *47*), thermal sensing (*6*, *48*), rotation sensing (*8*, *49*), and biomolecule detection (*22*). Achieving EP states involves meticulous parameter adjustments of the optical sensor, such as fine-tuning system coupling (*22*), adjusting gain-loss contrast (*6*), and changing the phase of mode coupling in the system (*20*). In other words, these optical structures serve a dual role, not only as sensors for detecting perturbations but also as crucial components in the realization of EPs. However, for widely adopted optical sensors like fiber-optic probes (*50*) and modularly packaged WGM sensors (*51*–*53*), dynamically manipulating their parameters to attain EPs is challenging and impractical. Meanwhile, it is worth noting that the majority of these conventional sensors inherently exhibit optical phase changes in response to detected targets or fields. Thus, it is essential to construct a configuration capable of enhancing optical phase changes via EPs, thereby benefiting various traditional optical sensors.

## RESULTS

**Fig. 1.**
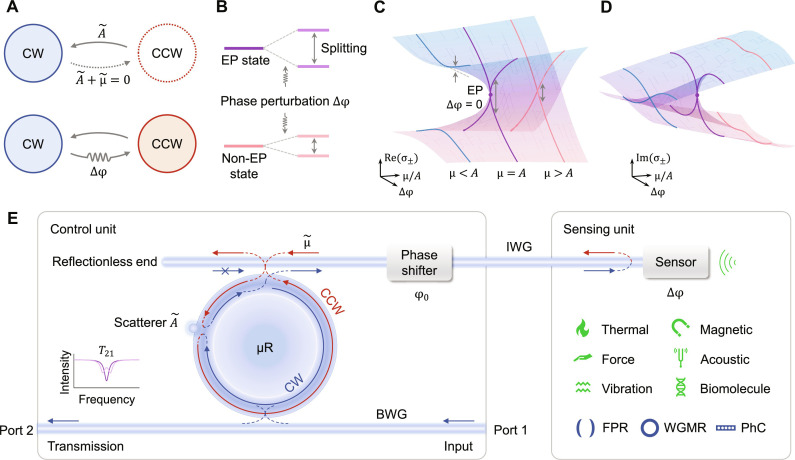
EP-enhanced phase sensing platform. (**A**) CW and CCW modes are coupled by bidirectional coupling channel $\tilde{A}$ and unidirectional coupling channel $\tilde{\mu }$ . At an EP state, one of the coupling directions (CW-to-CCW) is canceled by the destructive interference of two coupling channels, leading to the coalescence of two eigenmodes. As perturbed by an optical phase ∆φ, the system is detuned away from the EP state due to the recovered coupling channel, and its eigenstates are split (**B**). (B) Compared with non-EP states, the coalesced EP state has a more sensitive response to the phase perturbation ∆φ, embodied as a larger splitting of two eigenmodes. (**C** and **D**) Schematic topology of the surfaces that characterizes the real part (C) and imaginary part (D) of the complex eigenvalues σ_±_, indicating the frequency splitting and linewidth difference of two eigenmodes, respectively. In particular, the response to a sufficiently small perturbation ∆φ around an EP (μ/*A* = 1 and φ_0_ = α + π), labeled by purple points, is stronger than those at non-EP states. (**E**) The EP-enhanced phase sensing platform is composed of a control unit and a sensing unit. The control unit includes a WGM resonator, in which the bidirectional coupling channel $\tilde{A}$ and the unidirectional $\tilde{\mu }$ are achieved by a Rayleigh scatterer and a waveguide (IWG) connected with the sensing unit, respectively. The BWG monitors the states and spectra of this system. The sensing unit can be various types of optical sensors that return the perturbation of optical phase changes. The targets for detection encompass temperature fluctuations, magnetic fields, acoustic signals, etc. CW, clockwise; CCW, counterclockwise; BWG, bus waveguide; IWG, interconnected waveguide; FPR, Fabry-Pérot resonator; WGMR, whispering-gallery-mode resonator; PhC, photonic crystal.

In Fig. 1, we introduce an EP-enhanced sensing platform designed to accommodate a broad range of optical sensors. This system is partitioned into two components: a control unit and a sensing unit, due to the spatial separation between the EP resonator and the sensor (Fig. 1E). The sensing unit comprises sensors capable of changes in optical phase in response to perturbations induced by sensing targets or fields. The majority of conventional phase-sensitive optical sensors, such as WGMRs, Fabry-Pérot resonators (FPRs), PhCs, interferometers, and fiber grating sensors, can satisfy this requirement. The optical phase perturbed by the sensors is relayed to the control unit. Crucially, the EP states are realized by adjusting the control unit, obviating the need for additional parameter manipulation of the external sensor. Consequently, in principle, EP enhancement can be readily applied to conventional optical sensors with various applications through this platform.

Next, we implement the functionality of the EP control unit. A variety of approaches have been demonstrated to achieve EPs in the microresonators, where light propagates in a circular manner, such as inter-resonator mode coupling (*6*, *7*, *14*–*18*), surface scatterer steering (*19*–*21*), nonlinear-optic coupling (*8*, *9*), periodically modulated index (*13*), and asymmetric coupling by unidirectional waveguides (*10*–*12*, *18*, *54*, *55*). Here, we use an on-chip microtoroid resonator coupled with a bus waveguide (BWG) and an interconnected waveguide (IWG). BWG guides the input light and monitor the spectral characteristics, while IWG connects the sensing unit to the EP resonator through a phase shifter. The microresonator inherently supports clockwise (CW)– and counterclockwise (CCW)–traveling modes with degenerate eigenfrequency but orthogonal eigenvectors (*56*). To break the symmetry and steer the system toward an EP, we introduce a bidirectional coupling channel $\tilde{A}$ and a unidirectional coupling channel $\tilde{\mu }$ as shown in Fig. 1A. The effective 2 × 2 coupling Hamiltonian in the traveling-wave basis (CW and CCW) can be written as

$$H_{c}=\left(\begin{array}{cc} 0 & \tilde{A}\\ \tilde{A}+\tilde{\mu } & 0 \end{array}\right)$$ (1)

As shown in Fig. 1E, the bidirectional coupling $\tilde{A}$ ( = *Ae*^*i*α^) between the two traveling modes is enabled through isotropic scattering by an effective Rayleigh scatterer, such as nanoparticles or defects on the microtoroid surface. The coupling phase α is determined by the dissipative coupling $\left[\text{Im}\left(\tilde{A}\right)\right]$ of the scatterer. On the other hand, the unidirectional coupling $\tilde{\mu }$ ( = μ*e*^*i*φ^) is introduced by IWG, in which the CW mode can be coupled to the CCW via the reflection of the sensing unit, while the coupling from the CCW to the CW is prohibited due to the reflectionless design of one waveguide end. The coupling strength μ (= κ*r*) is affected by the resonator-IWG coupling strength κ as well as the reflectivity of the sensing unit *r*. The coupling phase φ is the sum of the phase offset φ_0_ controlled by the phase shifter and the phase perturbation ∆φ of the sensing unit. By solving the coupling Hamiltonian in Eq. 1, the bifurcation of eigenvalues is derived as

$$\sigma_{\pm }=\pm \sqrt{\tilde{A}\left[\tilde{A}+\mu \;\text{exp}\;i\left(\varphi_{0}+\Delta \varphi \right)\right]}$$ (2)

In the absence of perturbation (∆φ = 0), by carefully tuning the parameters into

$$\mu =A\;\text{and}\;\varphi_{0}=\alpha +\pi$$ (3)

the destructive interference cancels the CW-to-CCW coupling direction, i.e., $\tilde{A}+\tilde{\mu }=0$ . At this point, two eigenstates coalesce with the same eigenfrequency (σ_±_ = 0) and eigenvector, known as an EP. In practice, the conditions in Eq. 3 can be achieved by adjusting the resonator-IWG coupling distance to meet κ = *A*/*r*, while φ_0_ can be satisfied through tuning the phase shifter for any length of IWG. In principle, the system can reach EPs only by tuning the control unit without any sensor adjustments.

The real and imaginary parts of σ_±_ around the EP are exhibited by the topology of the eigensurfaces in Fig. 1 (C and D), representing the frequency splitting (2∣ Re (σ_±_)∣/2π) and linewidth difference (4∣ Im (σ_±_)∣/2π) between two eigenmodes, respectively. At the EP, both the frequency splitting and linewidth difference are zero (marked by purple points in Fig. 1, C and D). In response to a small phase perturbation ∆φ, a larger bifurcation (i.e., mode splitting) is observed near the EP state (μ = *A*) compared to non-EP states (μ ≠ *A*). This is because the change of spectral characteristics, e.g., frequency splitting, has a square-root relation to ∆φ at EPs (more details can be found in Materials and Methods and note S1). The enhanced responses at EPs are indicated by the arrows in Fig. 1 (B and C). The mode splitting can be measured from the transmission spectrum (*T*_21_) through BWG of the control unit.

### Approach EPs by tuning the control unit

Mode coalescence is a critical feature at EPs, which is a result of canceling the CW-to-CCW coupling channel by destructive interference in this configuration. In this subsection, we investigate the experimental criterion for finding and confirming EPs. The system is steered in parameter space by manipulating the coupling strength μ and phase φ_0_ of IWG. Conceptually, one of the two surface scatterers used in previous EP setups (*19*, *20*, *43*) is replaced by IWG, of which μ and φ_0_ are an analogy to the size and relative position of a scatterer, respectively.

**Fig. 2.**
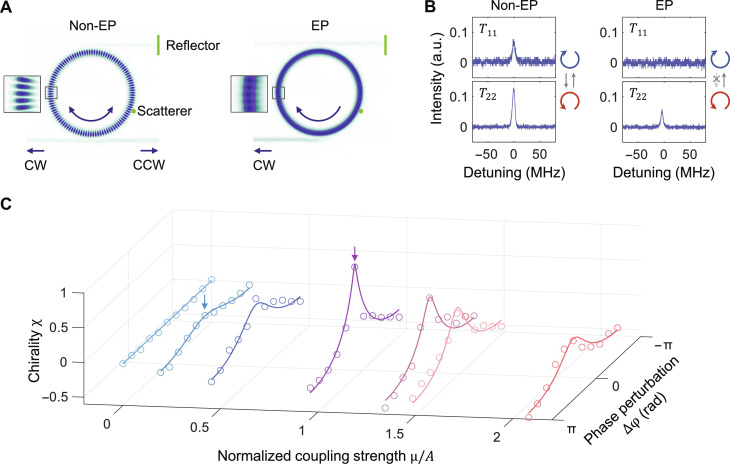
Realization of EPs. (**A**) The system at a non-EP state (left) supports both CW and CCW traveling modes in the resonator, and the simulated intracavity field pattern shows a standing wave solution. Because all the coupling channels exist, both CW and CCW modes can be excited regardless of the input mode, represented by the nonzero mode intensities (*T*_11,22_ ≠ 0) in (**B**, left). The system at an EP state (right) only has one eigenmode (CW mode in this configuration), and the simulation implies a unidirectional traveling wave solution. The canceled coupling channel blocks the excitation of the CCW mode when inputting the CW mode, i.e., zero mode intensity (*T*_11_ = 0) in (B, right). (B) Mode intensity spectra at the non-EP and EP states. *T_ij_*, input from port *j* and monitor the intensity from port *i*, as defined in Fig. 1. a.u., arbitrary units. (**C**) The measured chirality with regard to the coupling strength μ and the phase perturbation ∆φ. The chirality at EPs tends to the unity. The two arrow-labeled points correspond to the states in (A) and (B).

We verify the eigenmodes using a two-dimensional finite-element simulation (Fig. 2A), in which the coupling channels of the microresonator are modeled by a nanoparticle with a diameter of 100 nm and a unidirectional waveguide (IWG). One end of IWG is set as a reflector, while the other end extends to a perfectly matched layer to minimize reflection. BWG is used to extract the mode components (CW and CCW).

At a non-EP state (μ ≠ *A*), both CW and CCW modes coexist in the microresonator, and the overlapping of two modes results in a standing wave solution of the simulated intracavity field (Fig. 2A, left). Meanwhile, we experimentally investigate the coupling channels between CW and CCW modes (experiment setup in fig. S8). In this case, neither of the coupling directions is completely canceled because the CCW (CW) mode intensity does not vanish with the CW (CCW) input, i.e., nonzero mode spectra (*T*_11,22_ ≠ 0) shown in Fig. 2B (left). However, as the system is tuned to an EP, the system only supports a traveling wave (CW mode) as its eigenstate. The blurred field pattern implies a nearly pure traveling wave solution (Fig. 2A, right). In Fig. 2B (right), due to the canceled coupling direction (CW-to-CCW), the CCW intensity with CW input (*T*_11_) is zero. In contrast, the CW intensity with CCW input still exists (*T*_22_ ≠ 0) because the CCW-to-CW coupling is not canceled. Note that the spectra in the non-EP state do not exhibit the two split eigenmodes at this time, owing to the reduced resolvability caused by dissipation. It is worth emphasizing that the fundamental criterion for identifying EPs is the absence of a non-diagonal Hamiltonian element (Eq. 1), specifically, vanished CW-to-CCW coupling channel $\left(\tilde{A}+\tilde{\mu }=0\right)$ . This criterion serves as a guiding principle in finding and confirming EPs during the following experiments.

The asymmetric coupling, where one of the coupling directions is partially or completely canceled (*19*), results in nonzero chirality χ. The chirality is an intrinsic property only relying on the coupling symmetry and independent of the input field (see Materials and Methods). We sweep the coupling strength μ and phase ∆φ and record the reflection spectra for CW and CCW input directions (fig. S9). The nonzero chirality exists with asymmetric coupling (μ/*A* > 0) and particularly tends to unity (χ = +1) at the EP where the eigenstates coalesce into the CW mode (Fig. 2E). In the case of symmetric coupling (μ/*A* = 0), the system exhibits trivial chirality (χ = 0).

### Response of spectral characteristics

The design of this EP configuration enables us to convert optical phase change to the change in spectral characteristics, such as frequency splitting and linewidth difference. Before conducting measurements with an actual optical sensor, we investigate the spectral response of the system by an electrically controlled phase shifter to simulate the phase change induced by sensing targets. Note that the change of splitting when being perturbed by small phase is possibly obscured by broader mode linewidth due to the coupling dissipation (theoretical analysis found in Materials and Methods). To recognize the tiny spectral changes, we introduce erbium ions (2 × 10^19^ cm^−3^) into the silica microtoroid resonator, in which the optical gain provided by the erbium ions compensates the loss at 1550-nm band to resolve smaller splitting (*20*). The fabrication procedures have been described in previous work (*7*, *57*). We optimize the pump and probe powers as well as the coupling strength of BWG to make sure that the system works below lasing threshold and without any Fano-like line shape (*58*) that may disturb resolving the spectral responses (fig. S13).

**Fig. 3.**
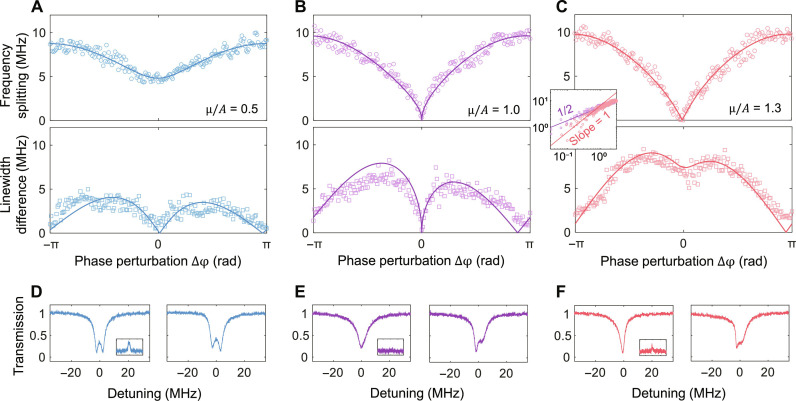
Characterization of the spectral responses to phase perturbations. (**A** to **C**) The frequency splitting and the linewidth difference as varying the phase perturbation ∆φ at three coupling regimes μ/*A*. At the EP state, frequency splitting, linewidth difference, and reflection [inset of (**E**)] are close to zero in experiments. The inset of (C) shows a logarithmic plot of frequency splitting. The small perturbations induce a square-root relation (slope = 1/2) at the EP case (μ/*A* = 1.0) and a linear relation (slope = 1) at the non-EP case (μ/*A* = 1.3), while they tend to be the same at larger perturbations. (**D** to **F**) The transmission spectra at ∆φ = 0 (left) and ∆φ = 0.5 (right). The changes of splitting at the EP are larger than the other two non-EP states. Insets: The reflection spectra at ∆φ = 0.

The frequency splitting and linewidth difference are extracted from the transmission spectra (*T*_21_) as we tune the phase perturbation ∆φ in the cases of μ < *A*, μ = *A*, and μ > *A* (Fig. 3, A to C). At the EP, both of them are nearly zero due to the mode coalescence. The experimental setup, measured spectra, and curve fitting can be found in figs. S10 to S12. The coupling strengths μ/*A* are obtained by fitting the frequency splitting according to the theoretical formula of Re(σ_±_). The frequency splitting has a square-root relation at μ = *A* and a linear relation at μ > *A* in response to small ∆φ, illustrated in the inset of Fig. 3C by the slope of 1/2 and 1, respectively, while both of them tend to saturate as ∆φ increases. Figure 3 (D to F) demonstrates the changes in transmission spectra from ∆φ = 0 to ∆φ = 0.5 for the three cases of μ/*A*, respectively. The change of frequency splitting at the EP is more notable than those at the non-EPs. On the basis of these findings, the sensitivity of an optical sensor is expected to be enhanced by connecting it to a control unit tuned at an EP state.

### Enhance strain sensing through an EP configuration

To demonstrate the universality of the proposed EP configuration that can be applied to any phase-sensitive sensor, we choose fiber strain as the detected target due to its straightforward relation to optical phase. Here, we use two types of fiber strain sensors as the sensing unit for demonstration, a reflection-type nonresonant sensor and a transmission-type resonant sensor.

**Fig. 4.**
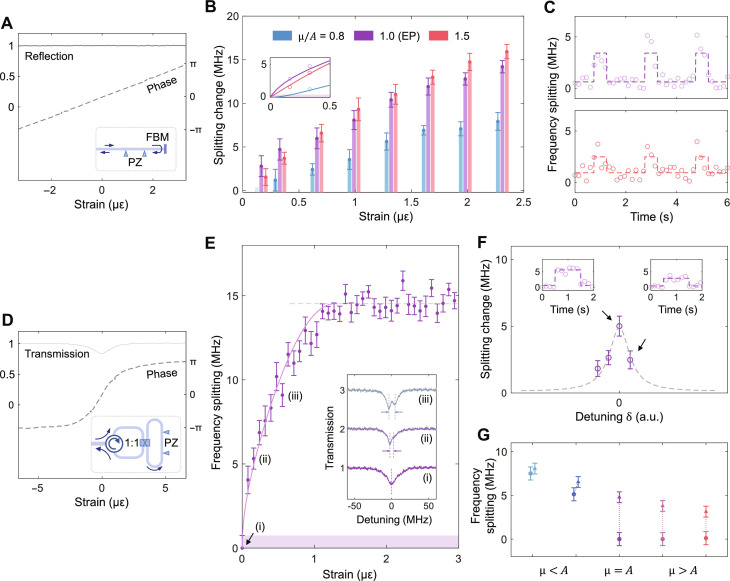
EP-enhanced fiber strain sensing. (**A**) Reflection-type nonresonant strain sensor. The PZ component stretches the fiber to introduce the optical phase change, and the FBM reflects the light to the EP control unit. The light goes through the stretched part double times. (**B**) The changes of frequency splitting as applying the pulsed strains in the cases of μ/*A* = 0.8, 1.0, and 1.5, respectively. The inset displays the fitted curves at smaller perturbations. The shaded area represents the fluctuation of the splitting at an EP. (**C**) The frequency splitting in response to the pulsed strains applied to the systems at the states of μ/*A* = 1.0 (top) and 1.5 (bottom). (**D**) Transmission-type resonant strain sensor. The fiber ring resonator is formed by connecting the ports of a 1:1 fiber splitter end to end. The light with phase change is sent back to the EP control unit by an optical circulator. (**E**) The frequency splitting at the EP state with regard to fiber strains. The splitting fluctuation is labeled by the shaded area. Inset: Typical transmission spectra. (**F**) The changes of frequency splitting with the strains applied at different detuning δ between the EP resonator and the fiber ring sensor. The dashed line represents the slope of the phase curve in (D). (**G**) The frequency splitting before (dots) and after (triangles) the strains applied at different μ. The maximum response occurs at an EP (μ = *A*). Data points for μ < *A* are plotted with lateral offsets to show error bars clearly. The amplitude of pulsed strains used in (C), (F), and (G) is 0.16 με. The unit of fiber strain (ε) is defined as the ratio of change in length to the original length. PZ, piezo; FBM: fiber-based mirror.

In the reflection-type nonresonant sensor (Fig. 4A), strain is introduced by stretching an 8-cm-long fiber using a piezo (PZ) component. The light is reflected by a fiber-based mirror (FBM) to the control unit, going through the stretched fiber twice and accumulating a total phase change ∆φ. The control unit is used to convert and amplify the ∆φ induced by the conventional strain sensor into a stronger frequency splitting. To reach an EP, the parameters of the control unit, including the resonator-IWG coupling distance and the offset φ_0_ of the phase shifter, are carefully adjusted to meet the demand in Eq. 3. Besides, the phase shifter is also modulated by a triangle waveform to compensate for the additional phase changes induced by the scanning of the probe light wavelength (see Materials and Methods).

We apply pulsed strains (period, 2 s; duty cycle, 25%) with different amplitudes to the fiber and record the transmission spectra at 0.15-s intervals. The changes in frequency splitting for three cases of μ/*A* = 0.8, 1.0, and 1.5 are plotted in Fig. 4B. The EP enhancement is more notable for small perturbations, e.g., < 0.5 με. The frequency splitting at μ = *A* and μ > *A* in response to the strain pulses is shown in Fig. 4C. The detection limit is defined as the strain that induces the splitting change equal to the splitting fluctuation, i.e., signal-to-noise ratio (SNR) = 1. The shaded area in the inset of Fig. 4B represents the splitting fluctuation near EPs (0.404 MHz). Thus, the detection limit at the EP is derived as 17 nε, which is diminished by 10.3 and 2.1 times than the other two non-EP cases, respectively.

The second strain sensor, a transmission-type resonant sensor, consists of a fiber ring resonator (Fig. 4D). Two ports of a 1:1 fiber splitter are connected for optical resonance. The perimeter of the ring is 56 cm, and the stretched length is the same as that of the first sensor (8 cm). The fiber ring resonator operates at an overcoupling state with a reduced variation in transmitted light intensity (∆*r* ~ 17%) (*59*), which, in turn, results in a lesser deviation from μ/*A* = 1 (μ = κ*r*, where κ is the coupling strength between IWG and the EP microresonator). This deliberate strategy mitigates the degradation of EP enhancement. The transmitted light with the phase change is sent back to the control unit through an optical circulator.

The behavior of the frequency splitting around an EP is measured when the fiber strains increase (Fig. 4E). The inset exhibits the examples of transmission spectra, where the fitted frequency splitting is labeled by arrows. The change of splitting is sensitive to the small strain perturbations and then saturates due to the smooth phase curve off resonance (Fig. 4D). The minimum detection limit at the EP is derived as 4.3 nε by SNR = 1 (the splitting fluctuation is 0.746 MHz). Furthermore, we investigate the splitting change at different detuning δ between the EP resonator and the fiber ring resonator (Fig. 4F). Note that the largest splitting change occurs at the matching point (δ = 0), where the sensing unit has the most sensitive phase response to strain due to the resonance of the fiber ring. In Fig. 4G, the arrows mark the change of frequency splitting at different coupling strengths and the maximum response occurs at the EP (μ = *A*).

In addition, the strain sensors are also characterized by a Mach-Zehnder interferometer with the same probe power (notes S2 and S3). The detection limits are derived as 57 nε and 25 nε for the reflection-type nonresonant sensor and transmission-type resonant sensor, respectively. The detection limits are reduced by 3.4 and 5.8 times as using the EP system. The origins of noise and the strategies to mitigate them are discussed in detail in note S4.

## DISCUSSION

In this configuration, EPs are achieved through the destructive interference of two coherent coupling channels. The phase change ∆φ of the unidirectional channel (reflective waveguide IWG, $\tilde{\mu }$ ) can be amplified around EPs, while the coupling phase α of the bidirectional channel (surface scatterer, $\tilde{A}$ ) can also influence the EP enhancement factor (details in note S1). The theoretical derivation shows that tuning α to π/4 can reduce the linewidth difference and increase the frequency splitting for a subtle phase perturbation Δφ ≪ 1. The EP enhancement factor will be further improved by $\sqrt{2}$ times.

The configuration with IWG brings unique flexibility to EP physics. Beyond changing the relative position of the scatterer, the manipulation of mode coupling has been achieved by adjusting the phase difference, reflectivity, and transmissivity in waveguide-based structures. This opens up possibilities for controlling EP states through various nonlinear optic effects, such as electro-optic, thermo-optic, and piezoelectric effects, offering enhanced feasibility and reliability. For example, the techniques of photonic integrated circuits, in which all the optical components are fabricated on a chip and the coupling strength/phase can be precisely tuned by electronics (*60*, *61*), will improve the stability and boost the performance of EP-enhanced sensing.

Furthermore, the EP enhancement of optical phase change is not limited to the demonstrated microresonator-waveguide platform. The tuning of the coupling phase can be extended to modulate the dispersion and dissipation of general coupling media. The phase-sensitive EP system can also be implemented in various structures, including but not limited to optical fibers, plasmonics, and metamaterials.

In summary, we have implemented a universal platform to enhance the sensing performance, including sensitivity and detection limit, leveraging the square-root topological features around EPs in response to phase perturbations. The platform can enable EP enhancement in various conventional optical sensors, such as WGMR, FPR, PhC, and fiber-based sensors. This universality is facilitated by a general physical quantity exploited in our EP configuration: the optical phase, which is present in almost all optical sensors as a response to sensing targets. In our system, a separate control unit operating at an EP eliminates the need for operating a conventional sensor toward an EP, simplifying its condition to achieve enhanced sensing performance. The demonstrated EP-enhanced phase sensing platform facilitates the implementation of non-Hermitian physics for optical sensing. Improving the accessibility of the EP-enhanced sensing, which is particularly valuable for detecting small phase perturbations, helps expand its impact across a wide range of applications, such as environmental monitoring, structural health diagnosis, and medical diagnosis.

## MATERIALS AND METHODS

### Hamiltonian and coupled mode equations

In the traveling-wave basis (CW and CCW), the effective 2 × 2 Hamiltonian is written as

$$H=H_{0}+H_{c}=\omega_{0}-i\frac{\Gamma_{0}}{2}+\left(\begin{array}{cc} 0 & \tilde{A}\\ \tilde{A}+\tilde{\mu } & 0 \end{array}\right)$$ (4)

with $\tilde{A}={\mathrm{Ae}}^{i\alpha }$ , $\tilde{\mu }=\mu e^{i\left(\varphi_{0}+\Delta \varphi \right)}$ , Γ_0_ = γ_0_ + κ_0_ + κ − *g*, and μ = κ*r*. Here, ω_0_ is the resonant frequency of the microtoroid without coupling channels, and γ_0_, κ_0_, and κ are the intrinsic loss, the coupling strength (loss) of BWG, and the coupling strength (loss) of IWG, respectively. The optical gain *g* is provided by erbium ions. *r* is the reflectivity of the sensing unit. $\tilde{A}$ $\left(\tilde{\mu }\right)$ is the complex bidirectional (unidirectional) coupling strength as described in Fig. 1. The splitting of the eigenmodes is solved as

$$\sigma_{\pm }\equiv \Omega \mp i\frac{\Gamma }{2}=\pm \sqrt{\tilde{A}\left(\tilde{A}+\tilde{\mu }\right)}$$ (5)

As the parameters are tuned to μ = *A* and φ_0_ = α + π, the coupling channel $\left(\tilde{A}+\tilde{\mu }\right)$ vanishes and the system is at an EP. If the phase perturbation is sufficiently small (Δφ ≪ 1), then the approximated splitting at the EP exhibits a marked change due to the square-root response to ∆φ

$$\sigma_{\pm }\approx \pm {\mathrm{Ae}}^{i\left(\alpha -\frac{\pi }{4}\right)}\sqrt{\Delta \varphi }$$ (6)

The detailed theoretical analysis is found in note S1. The linewidth needs to be sufficiently narrow to observe the frequency splitting in transmission spectra (*T*_21_), i.e., γ_0_ + κ_0_ + κ − *g* < 2 Re (σ_±_). The small phase perturbation gives σ_±_ → 0. Thus, the optical gain (*g* > 0) is required in the experiments for the splitting characterization. With the CW-direction input (*s*_in_), the coupled mode equations can be written as

$$i\frac{d}{\mathrm{dt}}a_{\text{CW}}=\left(-\Delta -i\frac{\Gamma_{0}}{2}\right)a_{\text{CW}}+\tilde{A}a_{\text{CCW}}+\sqrt{\kappa_{0}s_{\text{in}}}$$ (7)

$$i\frac{d}{\mathrm{dt}}a_{\text{CCW}}=\left(\tilde{A}+\tilde{\mu }\right)a_{\text{CW}}+\left(-\Delta -i\frac{\Gamma_{0}}{2}\right)a_{\text{CCW}}$$ (8)

The transmission expression used for curve fitting is obtained from the stationary solution $\frac{d}{\mathrm{dt}}a_{\text{CW}\left(\text{CCW}\right)}=0$

$$T_{21}=|1+i\kappa_{0}\frac{-\Delta -i\Gamma_{0}/2}{\left(-\Delta -i\Gamma_{0}/2+\Omega -i\Gamma /2\right)\left(-\Delta -i\Gamma_{0}/2-\Omega +i\Gamma /2\right)}{|}^{2}$$ (9)

### Derivation of the chirality

The chirality χ reveals the asymmetry of coupling channels, which can be characterized by the reflections as inputting the probe light from different directions (CW or CCW) (*19*)

$$R_{\text{CW}\;\text{input}}={|\kappa_{0}a_{\text{CCW}}|}^{2}={\left|\frac{\kappa_{0}\left(\tilde{A}+\tilde{\mu }\right)}{{\left(\Delta +i\Gamma_{0}/2\right)}^{2}-\tilde{A}\left(\tilde{A}+\tilde{\mu }\right)}\right|}^{2}$$ (10)

$$R_{\text{CCW}\;\text{input}}={|\kappa_{0}a_{\text{CW}}|}^{2}={\left|\frac{\kappa_{0}\tilde{A}}{{\left(\Delta +i\Gamma_{0}/2\right)}^{2}-\tilde{A}\left(\tilde{A}+\tilde{\mu }\right)}\right|}^{2}$$ (11)

The chirality is defined as

$$\chi =\frac{\sqrt{R_{\text{CCW}\;\text{inpu}t}-}\sqrt{R_{\text{CW}\;\text{input}}}}{\sqrt{R_{\text{CCW}\;\text{inpu}t}+}\sqrt{R_{\text{CW}\;\text{input}}}}\equiv \frac{1-B}{1+B}$$ (12)

where

$$B=\sqrt{\frac{R_{\text{CW}\;\text{input}}}{R_{\text{CCW}\;\text{input}}}}=\sqrt{1+2\frac{\mu }{A}\text{cos}\left(\varphi_{0}-\alpha \right)+{\left(\frac{\mu }{A}\right)}^{2}}$$ (13)

The symmetric coupling (μ = 0) gives a trivial chirality (χ = 0), while, at an EP, the complete cancelation of one coupling direction (μ/*A* = 1, φ_0_ = π + α) results in the chirality χ_EP_ = +1. The solid lines in Fig. 2 are fitted from the expression of χ.

### Compensation of additional phase changes induced by frequency scanning

In experiments, the output frequency of the tunable laser ω(*t*) is scanned back and forth to monitor the spectral characteristics (frequency splitting and linewidth difference). The phase offset φ*_L_* = ω(*t*)*L*/*c*, which is the phase change of the long fiber *L* due to the frequency scanning ω(*t*), cannot be neglected. One possible phenomenon is the deformation of transmission curves (fig. S14), because of the varying phase as scanning the laser output wavelength. Using a shorter fiber can reduce this effect (e.g., *L* < 0.5 m in Fig. 3). For the longer fiber used in the remote sensing experiments, a 4.98-kHz triangle signal with an optimized amplitude is applied to the phase shifter to compensate for the frequency-related additional optical phase. The derivation of EPs caused by laser scanning is avoided.

## Supplementary Materials

### This PDF file includes:

Supplementary Notes S1 to S4 Figs. S1 to S14 References
