# Supplementary material for: Exceptional–point–enhanced phase sensing
Source: Sci Adv. 2024 Apr 5;10(14):eadl5037. doi: 10.1126/sciadv.adl5037 (PMC10997194; doi:10.1126/sciadv.adl5037)
Supplement: Supplementary file 1 — Supplementary Notes S1 to S4 Figs. S1 to S14 References [file sciadv.adl5037_sm.pdf]

Science Advances
AAAS

Supplementary Materials for

Exceptional–point–enhanced phase sensing

Wenbo Mao *et al.*

Corresponding author: Lan Yang, yang@seas.wustl.edu

Sci. Adv. **10**, eadl5037 (2024)
DOI: 10.1126/sciadv.adl5037

This PDF file includes:

Supplementary Notes S1 to S4
Figs. S1 to S14
References

Supplementary Notes

1. Theoretical analysis of splitting

In the Methods section, we have solved the eigenvalues of the two-mode-coupled system as

$$\sigma_{\pm} \equiv \Omega \mp i\,\Gamma/2 = \pm\sqrt{\tilde{A}\left(\tilde{A}+\tilde{\mu}\right)} \tag{S1}$$

in which $\tilde{A}$ is the complex bidirectional coupling strength and $\tilde{\mu}$ is the complex unidirectional coupling strength (see Fig. 1 in the main text). The spectral characteristics, frequency splitting and linewidth difference, are defined as $2|\Omega|/2\pi$ and $2|\Gamma|/2\pi$, respectively. We plot them in fig. S1 at the coupling strengths μ/A = 0.5, 1.0, and 1.5, varying the phase $\Delta\varphi$. Both frequency splitting and linewidth difference reach zero simultaneously at the EP, where the eigenstates coalesce. As perturbed by a small phase $\Delta\varphi$, the change of splitting around the EP state ($\mu/A = 1$) is much larger than that at other states, showing EP-enhanced phase sensing. Note that a more remarkable EP enhancement is observed as perturbed by smaller phase changes (fig. S2), while the superiority of EPs degrades for larger perturbations, for example, $\Delta\varphi$ = 1.

The symmetry of the splitting curves is affected by the parameter α (fig. S3). When the bidirectional coupling strength $\tilde{A}$ is purely real, i.e., α = 0, the curves are symmetric about the y-axis ($\Delta\varphi$ = 0). However, the dissipative term of the coupling cannot be avoided since the scatterer also acts as a loss channel, leading to non-zero α. Our experimental results (Fig. 3 in the main text) display a slight asymmetry of the fitted curves. We have derived the splitting with $\Delta\varphi \ll 1$ in the Methods section, $\sigma_{\pm} \approx \pm A e^{i(\alpha-\pi/4)}\sqrt{\Delta\varphi}$. Assuming $\Delta\varphi > 0$, the frequency splitting, i.e., the real part of $\sigma_{\pm}$, is given as

$$2\left|\mathrm{Re}(\sigma_{\pm})\right| \approx 2A\left|\cos\left(\alpha-\frac{\pi}{4}\right)\right|\sqrt{\Delta\varphi} \tag{S2}$$

Theoretically, the maximum response to a small phase perturbation occurs at $\alpha = \pi/4$. The EP enhancement can be further improved $|\cos(0)|/|\cos(-\pi/4)| = \sqrt{2} \approx 1.4$ times than the current results. One may tune the α by a chromium-coated nanotip (*62*).

2. Reflection-type remote strain sensor

As described in the main text, the reflection-type sensor (RS) is constructed by a fiber stretched by a piezo (PZ) stage and a fiber-based mirror (FBM). The length of stretched fiber l is set as 8 cm. The movement of the PZ stage, driven by an arbitrary waveform generator and an amplifier, leads to an optical change induced by the fiber strain. The phase change is accumulated twice due to the reflection of the FBM. To avoid unwanted mechanical instability, all the components are fixed inside an acrylic box that is placed on a suspended optical table.

To compare with our EP-enhanced sensing, we first characterize the RS is using a Mach-Zehnder interferometer (MZI). The input power is the same as the probe power (2.5 μW) used in the EP sensing experiment. An optical circulator helps to connect the RS to one arm (fig. S4B). The output voltages of the photodetector (PD) are recorded as the strain varies in fig. S4C. The electric noise through a 20-MHz low-pass filter is 2.45 mV. The detection limit is derived to be 57 nε.

The response of the EP sensing system is shown in fig. S5, as perturbed by a pulsed strain, which has a period of 2 s, a duty cycle of 50%, and an amplitude of 1.32 με. A driving voltage is applied to the PZ stage to generate the fiber strain. Replacing the fragile tapered fibers with on-chip waveguides can reduce the splitting fluctuations caused by mechanical instability, thereby improving the detection limit of the EP sensing system.

The advantage of EP states, i.e., higher sensitivity, lies in their susceptibility to small phase changes. Fig. 4C in the main text shows that the frequency splitting at an EP is larger than that in the case of $\mu > A$, with the strain perturbation 0.16 με. However, for larger perturbations, for example 0.33 με, the magnitude of splitting at the EP ($\mu = A$) is very close to that at the non-EP ($\mu > A$) (fig. S6).

3. Transmission-type remote strain sensor

The transmission-type sensor (TS) is based on a fiber ring resonator, constructed from a 1:1 fiber splitter with the two interlinked ports externally connected. The theoretical expressions can be derived from the matrix method (*63*)

$$b_1 = \frac{-s + te^{-i\theta}}{-st + e^{-i\theta}} \quad \text{(S3)}$$

$$\text{Transmission} = |b_1|^2,\ \text{Phase} = \arg(b_1)$$

where t is the amplitude ratio of the beam splitter, s is the inner circulator factor, $\theta = \omega L/c$ represents the phase accumulation per circle, and L is the length of the fiber resonator. In our experiments, the fiber ring resonator works in the over-coupling regime to reduce the transmission variation with changing detuning (*59*). The transmission of the TS affects the unidirectional coupling strength μ, a critical parameter for EP tuning. The approach to introducing the fiber strain is the same as described in note S2.

The TS is characterized by an MZI, which consists of three fiber splitters and one fiber combiner (fig. S7B). The transmission and the phase response of the TS are monitored by PD1 and PD2, respectively, while PD3 is used to check the reference beam. fig. S7C displays the measured transmission and the phase change. The curve fitting gives t = 0.67, s = 0.98, and L = 0.56 m. The phase change is obtained from the interference pattern. The maximum slope is about 4 times larger than the phase change without the resonant structure (dashed line). The interference patterns (fig. S7D) vary with different phases of the reference beam ($-\varphi_r$). The detection limit (25 nε) is derived from the noise level and estimated at the maximum slope at $\varphi_r \approx \pi/2$.

4. Noise and mitigation strategies

In the main text, we have demonstrated the reduction of the detection limit by operating a sensor at EPs. The theoretical analysis reveals that the frequency splitting around a second-order EP scales as $\sqrt{\epsilon}$ and the enhancement factor $1/\sqrt{\epsilon}$ tends to infinity for a small perturbation $\epsilon \to 0$. However, EP-enhanced fiber strain sensing demonstrates a finite reduction in the detection limit. The detection limit of this EP platform is determined by the fluctuation of splitting, as quantified by SNR = 1. These experiments in classical regimes are far from the fundamental noise limitation caused by non-Hermitian eigen-basis collapse. Technical noise in classical systems, such as the vibration of optical and electric measurement components, dominates in monitoring the fluctuations of splitting at transmission spectra. Below are examples of sources that may impact the detection limit.

1) Mechanical instability that deviates the system from EPs: Examples include airflow and the vibration of the optical table affecting the gap between the tapered fibers and the microtoroid (resonator-IWG coupling μ), as well as the unwanted phase change in the unfixed fiber that connects the control and sensing unit (φ_0). Thermal drifts of the piezoelectric components in the translation stages or the phase shifter are also a source of mechanical instability.
2) Thermo-refractive noise in high-quality-factor (Q) microresonators: This can be suppressed by reducing optical powers and using a medium with compensated thermo-optic coefficients (*64*).
3) Drift in the tunable laser output: Variations in power and center wavelength can cause this.
4) Electric noise of the photodetector and the oscilloscope: This is particularly noticeable in the case of weak optical probe signals (several microwatts to nanowatts level) and can be mitigated by improving the photodetector responsivity or applying a low-pass filter to electrical signals.
5) Thermal fluctuations: These affect the resonant frequency of the microresonator or the phase offset of IWG (φ_0) and can be reduced by placing the components with a high-precise closed-loop temperature control unit.
6) Extraction errors in the case of small frequency splitting: The small frequency splitting may be overwhelmed by broad mode linewidths so that it cannot be resolved from the transmission spectra directly. Optical gain can be introduced by erbium-ion doping to compensate for the system dissipation, as described in Materials and Methods of the main text. Additionally, the accuracy and reliability of extracting tiny splitting (*e.g.*, sub-MHz) can be further improved by optimizing the curve fitting algorithm or with the assistance of machine-learning models.

Note that the robustness analysis (*65*) suggests that the EP system can be stabilized against weak parametric noise. Classical-noise-induced instability can, to some extent, be mitigated through technical methods, such as closed-loop control for drift compensation, lock-in amplifier for extracting signal obscured by various noises, and mechanically fixing fragile photonic structures. Despite the observed increase in noise at EPs, improved SNRs have been experimentally demonstrated in micromechanical (*66*) and electronic (*27*) systems.

In our experiments, the splitting fluctuation is dominated by mechanical instability. To minimize this fluctuation, we adopt the following strategies: a) The whole system is built on an actively damped optical table with pneumatic isolation; b) the EP control and sensing components are housed inside an acrylic box that is closed during experiments; c) two closed-loop piezo-electric nano-translation stages are used to precisely control the resonator-IWG coupling μ and the

resonator-BWG coupling; and d) the IWG fiber is fixed on the optical table to decrease the fluctuation of φ_0.

Looking ahead, EP enhancement can be further improved through integrated photonics techniques, where all the optical components are fabricated on the same chip to reduce mechanical instability. Besides, coupling strengths and phases can be precisely tuned by various mechanisms, such as the electro-optic (EO) effect of lithium niobate, the thermo-optic effect of silicon nitride, and the piezoelectric effect of aluminum nitride. For example, a variable coupling strength μ can be realized using a pair of 50:50 directional couplers and EO tunable optical paths, as demonstrated in Refs. (*60*, *67*). Dynamic control of EP states with stability and reliability can be achieved through high-speed EO modulation and feedback loops.

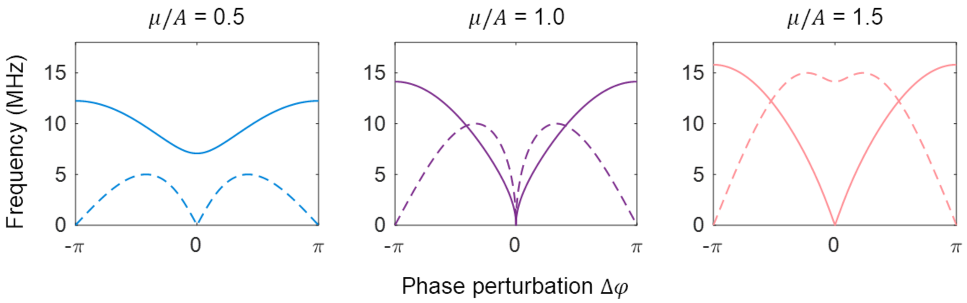

Fig. S1. Frequency splitting (solid lines) and linewidth difference (dashed lines) at different coupling strengths. The splitting exhibits more drastic change around the EP ($\mu/A = 1$). Parameters: $A/2\pi = 5$ MHz, $\alpha = 0$, $\varphi_0 = \pi$.

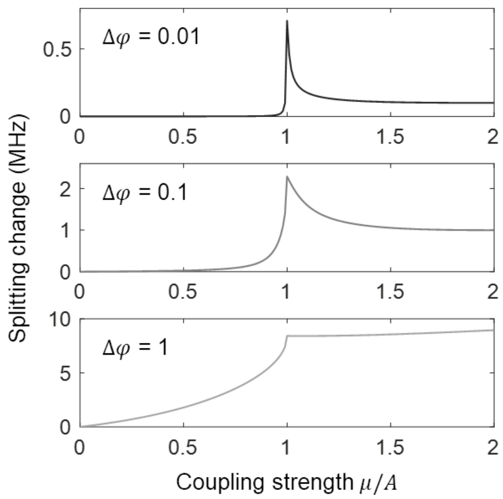

Fig. S2. Changes of splitting with a phase perturbation. The EP-enhancement is more significant for a smaller perturbation $\Delta\varphi$. With a larger $\Delta\varphi$ applied, the enhancement degrades since the changes of splitting at $\mu/A > 1$ could be larger than that at $\mu/A = 1$ (EP).

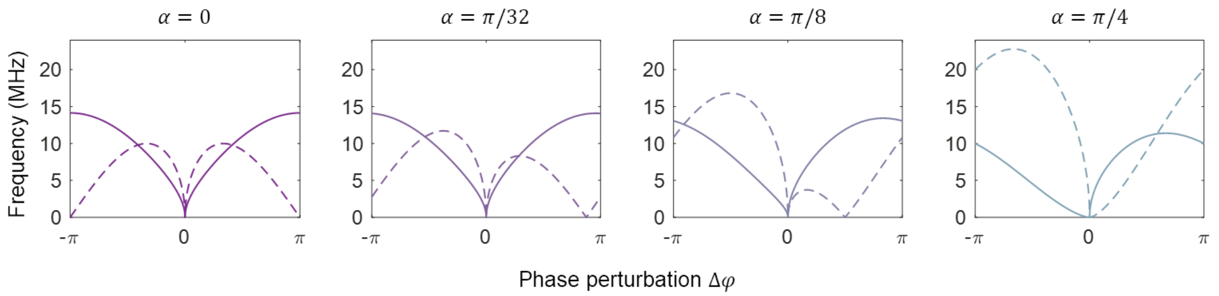

Fig. S3. Changes of the frequency splitting (solid lines) and linewidth difference (dashed lines) as the parameter α varies. The curves are symmetric about the y-axis in the case of $\alpha = 0$. With increasing α, the curves become asymmetric, and the change of frequency splitting at a sufficiently small phase perturbation ($0 < \Delta\varphi \ll 1$) also increases until reaching the maximum at $\alpha = \pi/4$. Parameters: $\mu = A = 2\pi \times 5$ MHz, $\varphi_0 = \pi + \alpha$.

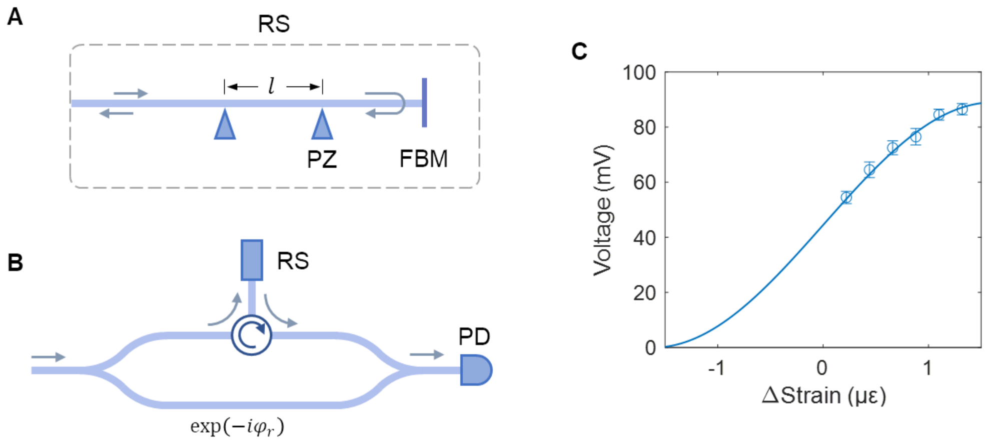

Fig. S4. Characterization of the reflection-type sensor. (**A**) Schematic of the RS for strain sensing. (**B**) Characterization of the RS with an MZI. The RS is connected to one arm by an optical circulator. (**C**) Measured signals as the applied strain increases when the reference phase φ_r is locked at $\pi/2$. RS, reflection-type sensor; PZ, piezo; FBM, fiber-based mirror; PD, photodetector.

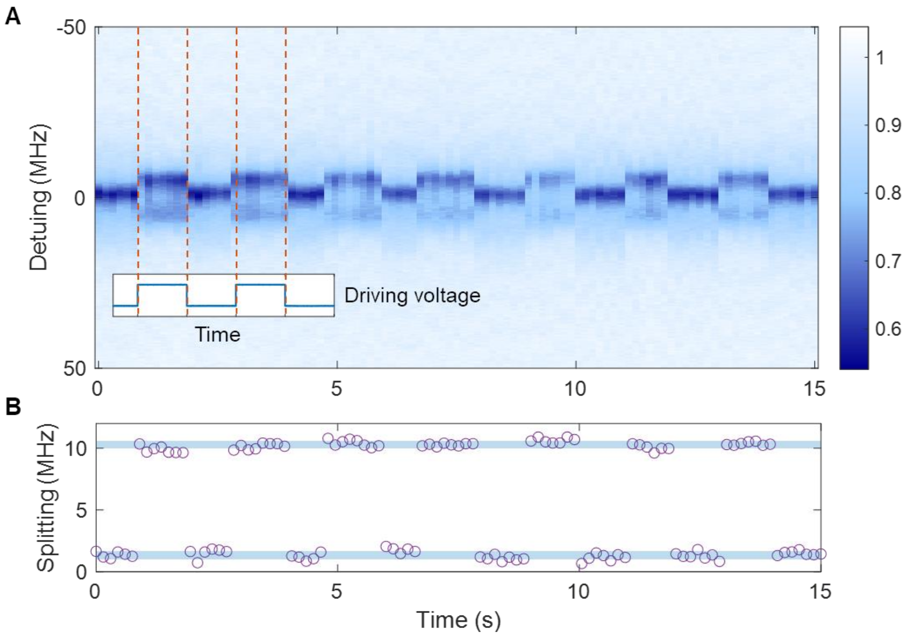

Fig. S5. Frequency splitting in response to a pulsed strain. (**A**) The system at an EP state is perturbed by a 2s 50% pulsed strain in the fiber (1.32 με). (**B**) Changes in frequency splitting when the system is perturbed by a pulsed strain. The fluctuations of the frequency splitting without and with strain in the fiber are 0.32 MHz and 0.31 MHz, respectively.

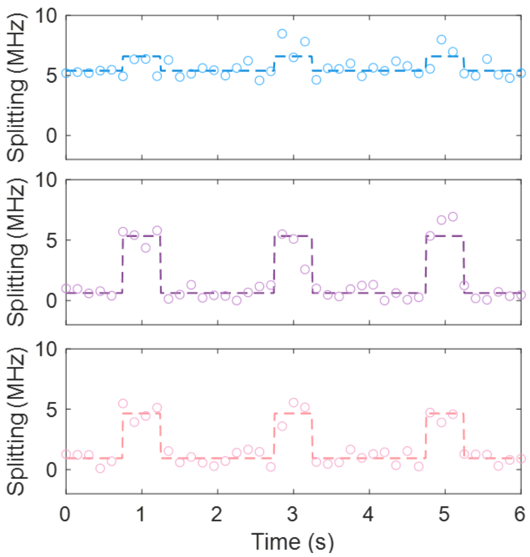

Fig. S6. Frequency splitting in response to a stronger pulsed strain. The amplitude of strains is 0.33 με. From top to bottom, the system is at the states $\mu < A$, $\mu = A$ (EP), and $\mu > A$, respectively. The EP enhancement is negligible for large perturbations.

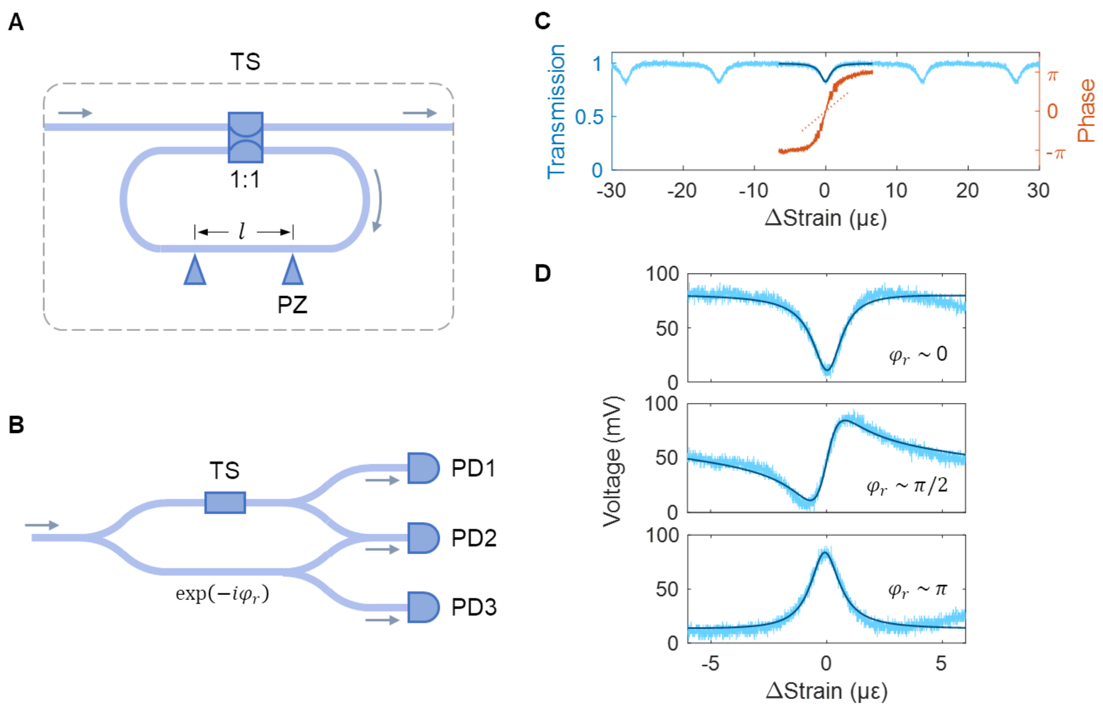

Fig. S7. Characterization of the transmission-type resonant strain sensor using an MZI. (**A**) Schematic of the fiber ring resonator for strain sensing. (**B**) Characterization of the TS using an MZI. The transmission (PD1) and phase (PD2) of the light through the TS, along with the transmission of the reference path (PD3), are monitored. (**C**) The measured transmission and phase change of the TS. Dashed line: the phase change without the resonant structure. (**D**) Signal output of PD2. Fitted φ_r/π = 0.04, 0.48, 0.98, respectively. TS, transmission-type sensor; PZ, piezo; PD, photodetector.

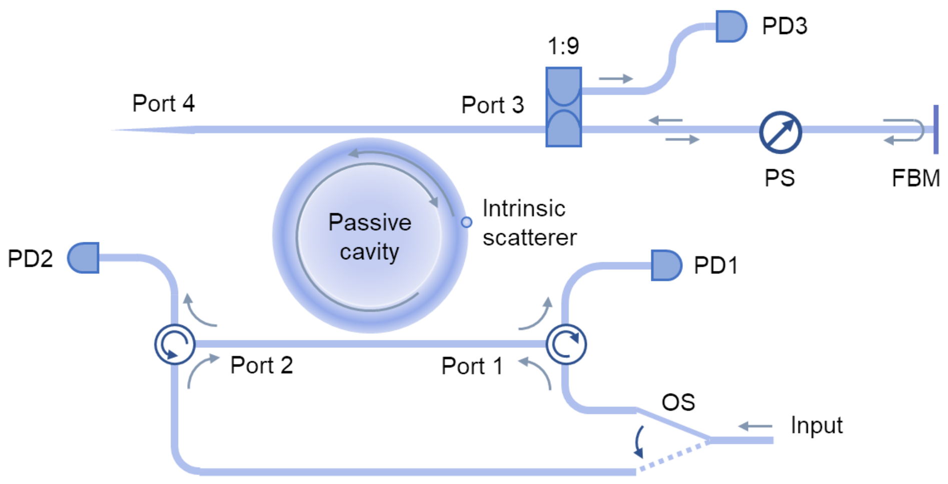

Fig. S8. Experimental setup for the chirality measurement. When inputting the CW mode, the transmission spectra are monitored by PD2, and the intensities of CCW and CW modes are measured by PD1 and PD3, respectively. For the CCW input direction, PD1 is used to monitor the transmission spectra, while both PD2 and PD3 measure the intensity of CW modes. PD, photodetector; PS, phase shifter; FBM, fiber-based mirror; OS, optical switch.

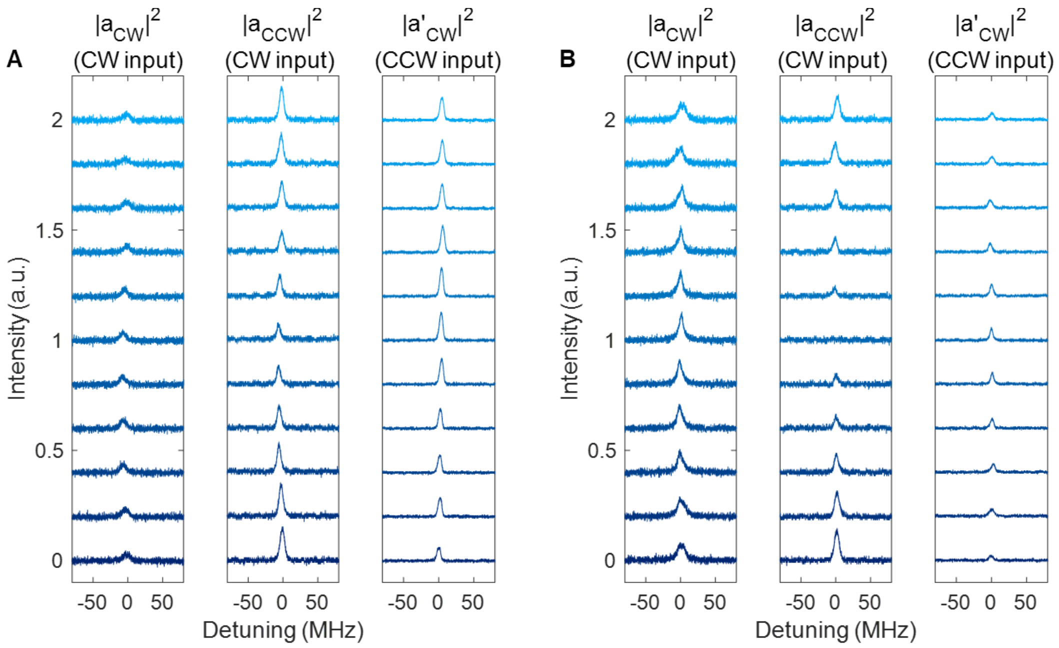

Fig. S9. Intensities of CW and CCW modes for different input directions. Vertical axis: varying $\Delta\varphi$ within $[-\pi, \pi]$. (A) $\mu < A$. (B) $\mu = A$. The reflection vanishes at the EP with the CW input direction.

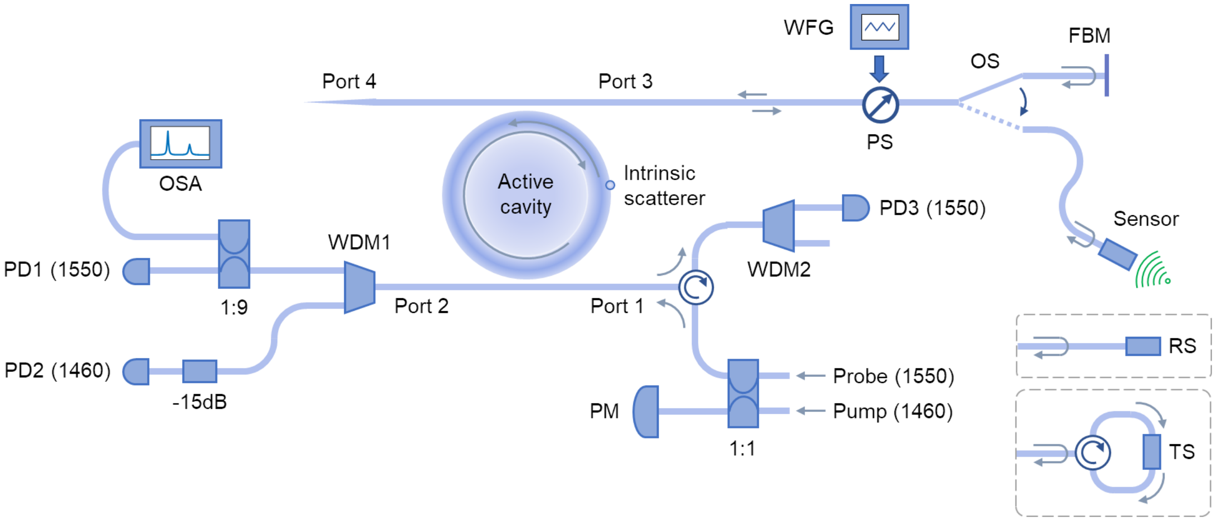

Fig. S10. Experimental setup for splitting measurements. The active cavity, pumped by the 1460-band light, provides optical gain for linewidth narrowing in the 1550-band probe light. PD1 and PD2 monitor the transmission spectra of the probe light and the pump light, respectively. The EP states are confirmed by zero reflections at PD3. The PS adjusts the phase offset φ_0 to realize EPs and compensate for additional frequency-related phase changes (Methods and fig. S14). Through the manual OS, port 3 of the control unit connects either to the FBM for the splitting characterization or to the remote sensor for sensing experiments. WFG, waveform generator; PS, phase shifter; OS, optical switch; FBM, fiber-based mirror; OSA, optical spectrum analyzer; WDM, wavelength-division multiplexing; PD, photodetector; PM, power meter; RS, reflection-type sensor; TS, transmission-type sensor.

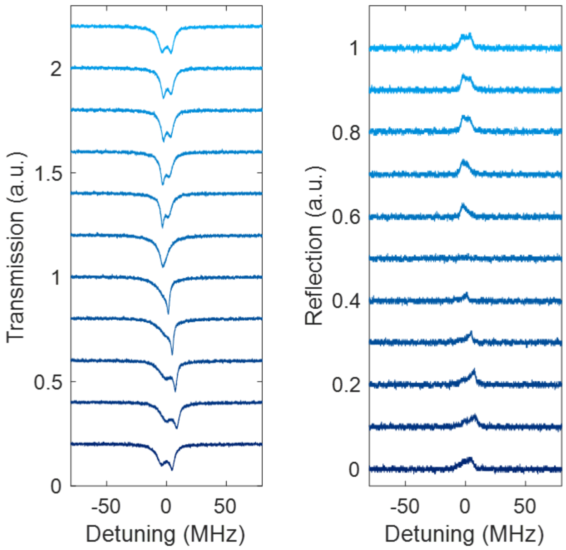

Fig. S11. Transmission and reflection spectra while tuning around an EP. From bottom to top, $\Delta\varphi$ varies from $-\pi$ to π. The EP state exhibits an unsplit transmission spectrum and a zero reflection.

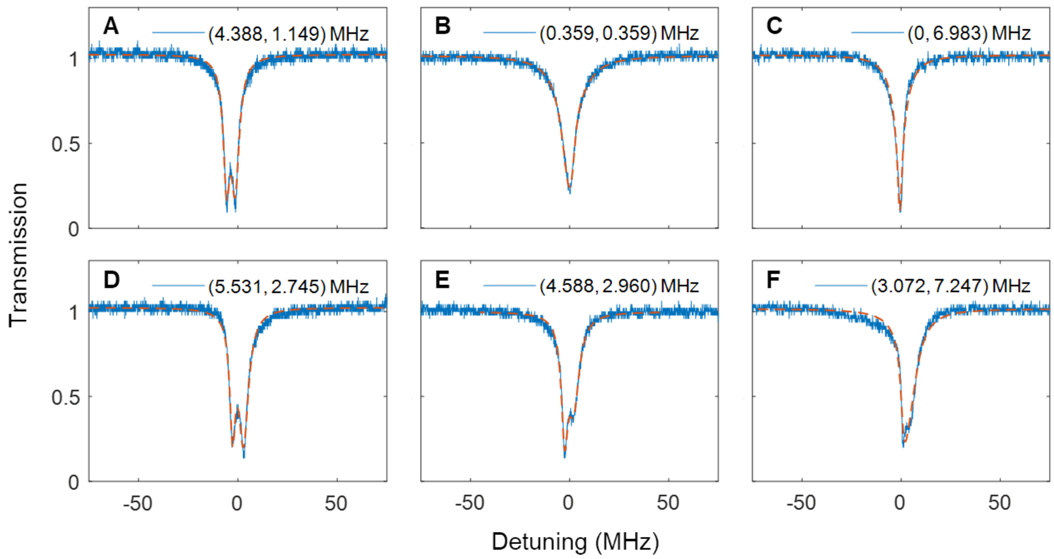

Fig. S12. Extraction of splitting from the transmission spectra. The curve fitting is implemented using the expression derived in the Methods section. Legend: (frequency splitting = $|2\Omega/2\pi|$, linewidth difference = $|2\Gamma/2\pi|$). (**A**, **B**, and **C**) μ/A = 0.5, 1.0, 1.3 for $\Delta\varphi$ = 0, respectively. (**D**, **E**, and **F**) μ/A = 0.5, 1.0, 1.3 for $\Delta\varphi$ = 0.5, respectively.

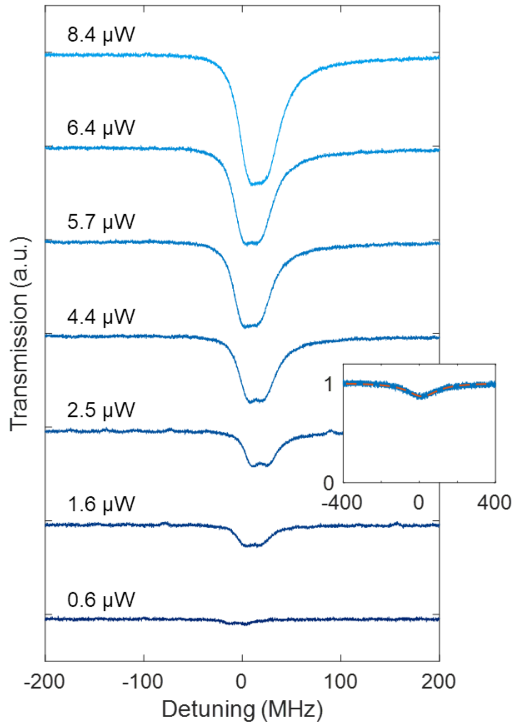

Fig. S13. Transmission spectra with increasing probe power. The probe light in the active cavity opens a lossy channel for the gain provided by the pump (*58*). Therefore, higher probe power compromises the ability to use optical gain to narrow the linewidth. Inset: Broader Lorentzian lineshape of the probe light without the pump light. Our system works in the under-coupling regime.

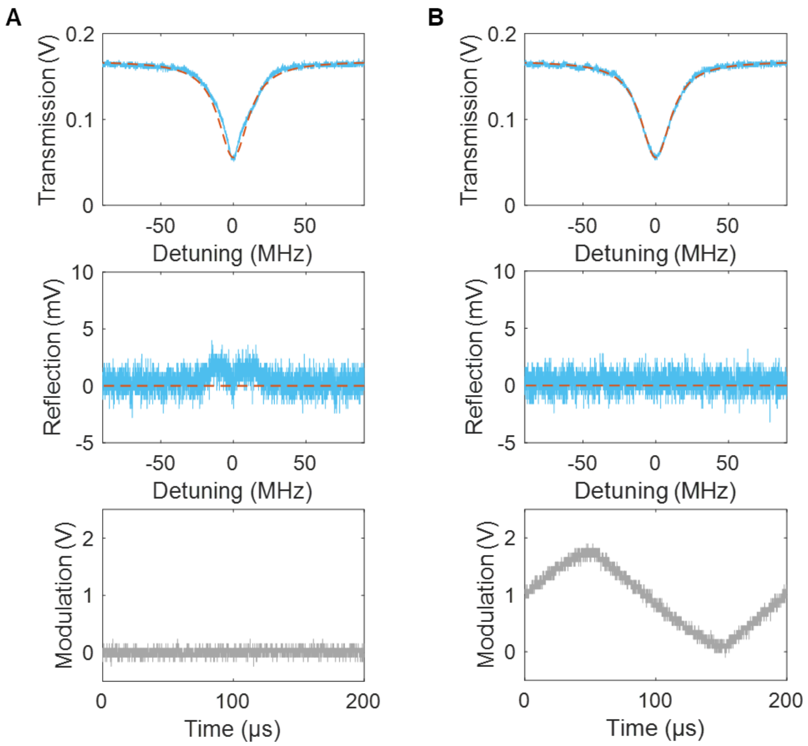

Fig. S14. Compensation of additional phase change induced by frequency scanning. (**A**) Without the modulation signal, the EP state is only achieved at zero detuning, which is confirmed by the zero reflection. The non-zero reflection elsewhere indicates the deviation from the EP state, because the frequency detuning leads to additional phase change (Materials and Methods). Also, the lineshape also deviates from the theoretical expectation. (**B**) With the modulation signal (4.98 kHz), the effect of the frequency-induced phase change is suppressed. The reflection remains zero at different detuning, and the lineshape of the transmission spectrum is recovered.

REFERENCES AND NOTES

1. K. Özdemir, S. Rotter, F. Nori, L. Yang, Parity–time symmetry and exceptional points in photonics. *Nat. Mater.* **18**, 783–798 (2019).

2. M. A. Miri, A. Alù, Exceptional points in optics and photonics. *Science* **363**, aar7709 (2019).

3. J. Wiersig, Review of exceptional point-based sensors. *Photonics Res.* **8**, 1457 (2020).

4. A. Li, H. Wei, M. Cotrufo, W. Chen, S. Mann, X. Ni, B. Xu, J. Chen, J. Wang, S. Fan, C.-W. Qiu, A. Alù, L. Chen, Exceptional points and non-Hermitian photonics at the nanoscale. *Nat. Nanotechnol.* **18**, 706–720 (2023).

5. C. Wang, Z. Fu, W. Mao, J. Qie, A. D. Stone, L. Yang, Non-Hermitian optics and photonics: From classical to quantum. *Adv. Opt. Photonics.* **15**, 442 (2023).

6. H. Hodaei, A. U. Hassan, S. Wittek, H. Garcia-Gracia, R. El-Ganainy, D. N. Christodoulides, M. Khajavikhan, Enhanced sensitivity at higher-order exceptional points. *Nature* **548**, 187–191 (2017).

7. B. Peng, Ş. K. Özdemir, F. Lei, F. Monifi, M. Gianfreda, G. L. Long, S. Fan, F. Nori, C. M. Bender, L. Yang, Parity–time-symmetric whispering-gallery microcavities. *Nat. Phys.* **10**, 394–398 (2014).

8. Y. H. Lai, Y. K. Lu, M. G. Suh, Z. Yuan, K. Vahala, Observation of the exceptional-point-enhanced Sagnac effect. *Nature* **576**, 65–69 (2019).

9. F. Zhang, Y. Feng, X. Chen, L. Ge, W. Wan, Synthetic anti-PT symmetry in a single microcavity. *Phys. Rev. Lett.* **124**, 053901 (2020).

10. Q. Zhong, J. Ren, M. Khajavikhan, D. N. Christodoulides, K. Özdemir, R. El-Ganainy, Sensing with exceptional surfaces in order to combine sensitivity with robustness. *Phys. Rev. Lett.* **122**, 153902 (2019).

11. S. Soleymani, Q. Zhong, M. Mokim, S. Rotter, R. El-Ganainy, Ş. K. Özdemir, Chiral and degenerate perfect absorption on exceptional surfaces. *Nat. Commun.* **13**, 1–8 (2022).

12. K. Liao, Y. Zhong, Z. Du, G. Liu, C. Li, X. Wu, C. Deng, C. Lu, X. Wang, C. T. Chan, Q. Song, S. Wang, X. Liu, X. Hu, Q. Gong, On-chip integrated exceptional surface microlaser. *Sci. Adv.* **9** (2023), doi:10.1126/sciadv.adf3470, eadf3470.

13. L. Feng, Z. J. Wong, R. M. Ma, Y. Wang, X. Zhang, Single-mode laser by parity-time symmetry breaking. *Science* **346**, 972–975 (2014).

14. L. Chang, X. Jiang, S. Hua, C. Yang, J. Wen, L. Jiang, G. Li, G. Wang, M. Xiao, Parity-time symmetry and variable optical isolation in active-passive- coupled microresonators. *Nat. Photonics.* **8**, 524–529 (2014).

15. H. Hodaei, M. Mohammad-Ali, M. Heinrich, D. N. Christodoulides, M. Khajavikhan, Parity-time-symmetric microring lasers. *Science* **346**, 975–978 (2014).

16. A. Tikan, J. Riemensberger, K. Komagata, S. Hönl, M. Churaev, C. Skehan, H. Guo, R. N. Wang, J. Liu, P. Seidler, T. J. Kippenberg, Emergent nonlinear phenomena in a driven dissipative photonic dimer. *Nat. Phys.* **17**, 604–610 (2021).

17. C. Wang, W. R. Sweeney, A. D. Stone, L. Yang, Coherent perfect absorption at an exceptional point. *Science* **373**, 1261–1265 (2021).

18. Q. Zhong, J. Kou, S. K. Ozdemir, R. El-Ganainy, Hierarchical construction of higher-order exceptional points. *Phys. Rev. Lett.* **125**, 203602 (2020).

19. B. Peng, Ş. K. Özdemir, M. Liertzer, W. Chen, J. Kramer, H. Yilmaz, J. Wiersig, S. Rotter, L. Yang, Chiral modes and directional lasing at exceptional points. *Proc. Natl. Acad. Sci. U.S.A.* **113**, 6845–6850 (2016).

20. W. Chen, Ş. K. Özdemir, G. Zhao, J. Wiersig, L. Yang, Exceptional points enhance sensing in an optical microcavity. *Nature* **548**, 192–196 (2017).

21. H. Lee, A. Kecebas, F. Wang, L. Chang, S. K. Özdemir, T. Gu, Chiral exceptional point and coherent suppression of backscattering in silicon microring with low loss Mie scatterer. *eLight.* **3** (2023), doi:10.1186/s43593-023-00043-5.

22. J. H. Park, A. Ndao, W. Cai, L. Hsu, A. Kodigala, T. Lepetit, Y. H. Lo, B. Kanté, Symmetry-breaking-induced plasmonic exceptional points and nanoscale sensing. *Nat. Phys.* **16**, 462–468 (2020).

23. Q. Song, M. Odeh, P. Genevet, Plasmonic topological metasurface by encircling an exceptional point. *Science* **373**, 1133–1137 (2021).

24. B. Zhen, C. W. Hsu, Y. Igarashi, L. Lu, I. Kaminer, A. Pick, S. L. Chua, J. D. Joannopoulos, M. Soljačić, Spawning rings of exceptional points out of Dirac cones. *Nature* **525**, 354–358 (2015).

25. H. Zhou, C. Peng, Y. Yoon, C. W. Hsu, K. A. Nelson, L. Fu, J. D. Joannopoulos, M. Solja, B. Zhen, Observation of bulk Fermi arc and polarization half charge from paired exceptional points. *Science* **359**, 1009–1012 (2018).

26. H. Z. Chen, T. Liu, H. Y. Luan, R. J. Liu, X. Y. Wang, X. F. Zhu, Y. B. Li, Z. M. Gu, S. J. Liang, H. Gao, L. Lu, L. Ge, S. Zhang, J. Zhu, R. M. Ma, Revealing the missing dimension at an exceptional point. *Nat. Phys.* **16**, 571–578 (2020).

27. Z. Dong, Z. Li, F. Yang, C. W. Qiu, J. S. Ho, Sensitive readout of implantable microsensors using a wireless system locked to an exceptional point. *Nat. Electron.* **2**, 335–342 (2019).

28. L. Shao, W. Mao, S. Maity, N. Sinclair, Y. Hu, L. Yang, M. Lončar, Non-reciprocal transmission of microwave acoustic waves in nonlinear parity–time symmetric resonators. *Nat. Electron.* **3**, 267–272 (2020).

29. Z. Li, C. Li, Z. Xiong, G. Xu, Y. R. Wang, X. Tian, X. Yang, Z. Liu, Q. Zeng, R. Lin, Y. Li, J. K. W. Lee, J. S. Ho, C. W. Qiu, Stochastic exceptional points for noise-assisted sensing. *Phys. Rev. Lett.* **130**, 227201 (2023).

30. M. Yang, L. Zhu, Q. Zhong, R. El-Ganainy, P. Y. Chen, Spectral sensitivity near exceptional points as a resource for hardware encryption. *Nat. Commun.* **14**, 1–10 (2023).

31. R. Kononchuk, J. Cai, F. Ellis, R. Thevamaran, T. Kottos, Exceptional-point-based accelerometers with enhanced signal-to-noise ratio. *Nature* **607**, 697–702 (2022).

32. A. Suntharalingam, L. Fernández-Alcázar, R. Kononchuk, T. Kottos, Noise resilient exceptional-point voltmeters enabled by oscillation quenching phenomena. *Nat. Commun.* **14**, 5515 (2023).

33. A. Gupta, A. Kurnosov, T. Kottos, R. Thevamaran, Reconfigurable enhancement of actuation forces by engineered losses in non-Hermitian metamaterials. *Extrem. Mech. Lett.* **59**, 101979 (2023).

34. V. Domínguez-Rocha, R. Thevamaran, F. M. Ellis, T. Kottos, Environmentally induced exceptional points in elastodynamics. *Phys. Rev. Appl.* **13**, 1 (2020).

35. M. Naghiloo, M. Abbasi, Y. N. Joglekar, K. W. Murch, Quantum state tomography across the exceptional point in a single dissipative qubit. *Nat. Phys.* **15**, 1232–1236 (2019).

36. L. Feng, Y. L. Xu, W. S. Fegadolli, M. H. Lu, J. E. B. Oliveira, V. R. Almeida, Y. F. Chen, A. Scherer, Experimental demonstration of a unidirectional reflectionless parity-time metamaterial at optical frequencies. *Nat. Mater.* **12**, 108–113 (2013).

37. Z. Lin, H. Ramezani, T. Eichelkraut, T. Kottos, H. Cao, D. N. Christodoulides, Unidirectional invisibility induced by PT-symmetric periodic structures. *Phys. Rev. Lett.* **106**, 1–4 (2011).

38. M. Kim, K. Kwon, J. Shim, Y. Jung, K. Yu, Partially directional microdisk laser with two Rayleigh scatterers. *Opt. Lett.* **39**, 2423 (2014), 2426.

39. Z. Lin, A. Pick, M. Lončar, A. W. Rodriguez, Enhanced spontaneous emission at third-order dirac exceptional points in inverse-designed photonic crystals. *Phys. Rev. Lett.* **117**, 1–6 (2016).

40. L. Ferrier, P. Bouteyre, A. Pick, S. Cueff, N. H. M. Dang, C. Diederichs, A. Belarouci, T. Benyattou, J. X. Zhao, R. Su, J. Xing, Q. Xiong, H. S. Nguyen, Unveiling the enhancement of spontaneous emission at exceptional points. *Phys. Rev. Lett.* **129**, 1–7 (2022).

41. Q. Zhong, A. Hashemi, K. Özdemir, R. El-Ganainy, Control of spontaneous emission dynamics in microcavities with chiral exceptional surfaces. *Phys. Rev. Res.* **3**, 1–7 (2021).

42. M. Khanbekyan, J. Wiersig, Decay suppression of spontaneous emission of a single emitter in a high-Qcavity at exceptional points. *Phys. Rev. Res.* **2**, 1–6 (2020).

43. C. Wang, X. Jiang, G. Zhao, M. Zhang, C. W. Hsu, B. Peng, A. D. Stone, L. Jiang, L. Yang, Electromagnetically induced transparency at a chiral exceptional point. *Nat. Phys.* **16**, 334–340 (2020).

44. W. R. Sweeney, C. W. Hsu, S. Rotter, A. D. Stone, Perfectly absorbing exceptional points and chiral absorbers. *Phys. Rev. Lett.* **122**, 93901 (2019).

45. M. Parto, Y. G. N. Liu, B. Bahari, M. Khajavikhan, D. N. Christodoulides, Non-Hermitian and topological photonics: Optics at an exceptional point. *Nanophotonics.* **10**, 415–435 (2021).

46. H. Wang, X. Zhang, J. Hua, D. Lei, M. Lu, Y. Chen, Topological physics of non-Hermitian optics and photonics: A review. *J. Opt.* **23** (2021), doi:10.1088/2040-8986/ac2e15, 123001.

47. G. Q. Qin, R. R. Xie, H. Zhang, Y. Q. Hu, M. Wang, G. Q. Li, H. Xu, F. Lei, D. Ruan, G. L. Long, Experimental realization of sensitivity enhancement and suppression with exceptional surfaces. *Laser Photonics Rev.* **15**, 1–6 (2021).

48. H. Zhao, Z. Chen, R. Zhao, L. Feng, Exceptional point engineered glass slide for microscopic thermal mapping. *Nat. Commun.* **9**, 1–8 (2018).

49. M. P. Hokmabadi, A. Schumer, D. N. Christodoulides, M. Khajavikhan, Non-Hermitian ring laser gyroscopes with enhanced Sagnac sensitivity. *Nature* **576**, 70–74 (2019).

50. J. A. Guggenheim, J. Li, T. J. Allen, R. J. Colchester, S. Noimark, O. Ogunlade, I. P. Parkin, I. Papakonstantinou, A. E. Desjardins, E. Z. Zhang, P. C. Beard, Ultrasensitive plano-concave optical microresonators for ultrasound sensing. *Nat. Photonics.* **11**, 714–719 (2017).

51. X. Xu, W. Chen, G. Zhao, Y. Li, C. Lu, L. Yang, Wireless whispering-gallery-mode sensor for thermal sensing and aerial mapping. *Light Sci. Appl.* **7**, 62 (2018).

52. J. Zhu, G. Zhao, I. Savukov, L. Yang, Polymer encapsulated microcavity optomechanical magnetometer. *Sci. Rep.* **7**, 8896 (2017).

53. T. Tan, Z. Yuan, H. Zhang, G. Yan, S. Zhou, N. An, B. Peng, G. Soavi, Y. Rao, B. Yao, Multispecies and individual gas molecule detection using Stokes solitons in a graphene over-modal microresonator. *Nat. Commun.* **12**, 8–15 (2021).

54. M. De Carlo, F. De Leonardis, R. A. Soref, V. M. N. Passaro, Design of an exceptional-surface-enhanced silicon-on-insulator optical accelerometer. *J. Light. Technol.* **39**, 5954–5961 (2021).

55. S. Jiang, J. Li, Z. Li, Z. Li, W. Li, X. Huang, H. Zhang, G. Zhang, A. Huang, Z. Xiao, Experimental realization of exceptional surfaces enhanced displacement sensing with robustness. *Appl. Phys. Lett.* **123** (2023), doi:10.1063/5.0171249.

56. J. Zhu, S. K. Ozdemir, Y. F. Xiao, L. Li, L. He, D. R. Chen, L. Yang, On-chip single nanoparticle detection and sizing by mode splitting in an ultrahigh-Q microresonator. *Nat. Photonics.* **4**, 46–49 (2010).

57. L. He, Ş. K. Özdemir, J. Zhu, W. Kim, L. Yang, Detecting single viruses and nanoparticles using whispering gallery microlasers. *Nat. Nanotechnol.* **6**, 428–432 (2011).

58. F. Lei, B. Peng, Ş. K. Özdemir, G. L. Long, L. Yang, Dynamic Fano-like resonances in erbium-doped whispering-gallery-mode microresonators. *Appl. Phys. Lett.* **105**, 101112 (2014).

59. G. Liang, H. Huang, A. Mohanty, M. C. Shin, X. Ji, M. J. Carter, S. Shrestha, M. Lipson, N. Yu, Robust, efficient, micrometre-scale phase modulators at visible wavelengths. *Nat. Photonics.* **15**, 908–913 (2021).

60. W. Liu, M. Li, R. S. Guzzon, E. J. Norberg, J. S. Parker, M. Lu, L. A. Coldren, J. Yao, An integrated parity-time symmetric wavelength-tunable single-mode microring laser. *Nat. Commun.* **8**, 1–6 (2017).

61. H. Du, X. Zhang, C. G. Littlejohns, D. T. Tran, X. Yan, M. Banakar, C. Wei, D. J. Thomson, G. T. Reed, Nonconservative coupling in a passive silicon microring resonator. *Phys. Rev. Lett.* **124**, 2–7 (2020).

62. B. Peng, Ş. K. Özdemir, S. Rotter, H. Yilmaz, M. Liertzer, F. Monifi, C. M. Bender, F. Nori, L. Yang, Loss-induced suppression and revival of lasing. *Science* **346**, 328–332 (2014).

63. A. Yariv, Universal relations for coupling of optical power between microresonators and dielectric waveguides. *Electron. Lett.* **36**, 321–322 (2000).

64. L. He, Y. F. Xiao, C. Dong, J. Zhu, V. Gaddam, L. Yang, Compensation of thermal refraction effect in high-Q toroidal microresonator by polydimethylsiloxane coating. *Appl. Phys. Lett.* **93**, 201102 (2008).

65. J. Wiersig, Robustness of exceptional-point-based sensors against parametric noise: The role of Hamiltonian and Liouvillian degeneracies. *Phys. Rev. A* **101**, 1–9 (2020).

66. M. N. Zhang, L. Dong, L. F. Wang, Q. A. Huang, Exceptional points enhance sensing in silicon micromechanical resonators. *Microsyst. Nanoeng.* **10**, 12 (2024).

67. D. Jia, R. Zhang, C. Yang, Z. Hao, X. Yu, F. Gao, F. Bo, G. Zhang, J. Xu, Electrically tuned coupling of lithium niobate microresonators. *Opt. Lett.* **48**, 2744– 2747 (2023).
